# Tetanus

**Published:** 1879-03

**Authors:** 


### TETANUS.

From a very exhaustive "Study of Four Hundred and Fifteen cases of Tetanus" by D. W. Yandell, M. D., published in *Brain.* Part iii., we obtain the following points of interest:

*Sex.*—80 per cent, of cases in which sex was stated were males. The greater exposure of men to the exciting causes of tetanus is usually regarded as sufficieut to explain this disparity, yet in 34 cases of idiopathic tetanus 70 per cent. were males. Of males 44 per cent. recovered; of females 58 per cent; or, excluding puerpral tetanus 63 per cent recovered. These results approximate those furnished by Curling, while they differ from those stated by Poland (in Holmes' System of Surgery), who finds a much greater preponderance of cases in males and of deaths in females.

*Age.*—The first decade of life furnishes 7 per cent of all the cases, and of these 62 per cent died. If prognosis in tetanus be influenced by age it is less favorable at this period than between ten and twenty years, where the cases rose to 20 per cent while the mortality fell to 38 per cent. Owing to the large number of cases reported simply as adults, the death rate could not be made out in the remaining decades. Fifteen cases are recorded as occurring in individuals over fifty years and one case in a man aged eighty-nine is reported.

The tables furnish nothing new as to the character of the wounds which are succeeded by tetanus. Wounds of the extremeties and punctured wounds still cut the largest figures in this class of injuries. The popular belief that injuries of the foot are more liable than those of other parts to be followed by tetanus is quite confirmed as to punctured wounds in this situation—the large majority being inflicted by nails run into the foot.

*Date of Invasion.*—A much more important point is the time at which the tetanic symptoms appear. The disease supervened within two weeks after injury in one hundred and ninety-six cases. Of these 62.5 per cent died. From the 14th to the 21st day, 17 per cent of eighty-one cases were fatal. From the twenty-first to the forty-fourth day there were seventeen cases of which 17 per cent died. In cases occurring within seventy-two hours after injury, recoveries exceeded deaths as fourteen to eleven. In such as occurred between the fourth and ninth day deaths exceeded recoveries as seven to three. But where tetanic symptoms were delayed till the fourteenth day, recoveries were notably in excess of deaths—23 per cent.

*Duration of Symptoms.*—The tables confirm in the main the well-known aphorism of Hippocrates that tetanus, when fatal, is nearly always so within the first four days after the attack. The longest duration of symptoms is two-hundred and forty days; the shortest ending in recovery was one which had as its only symptom a slight trismus which disappeared after a little hypodermic atropia and can not fairly be regarded as a case of tetanus, The shortest ending in death is in a few minutes. Nine cases lasted forty-two days; eighteen cases lasted thirty days. Between these periods we find nineteen cases; sixty and ninety days are also represented.

*The Pulse and Temperature* have been recorded in too small a number of instances to admit any deductions.

*Treatmeut* has been so varied and complicated as to set at defiance all efforts at accurate tabulation. Selecting the fifty agents to which recoveries are credited, it will be found that in a very small number has the treatment been trusted to a single agent alone. In what follows, the writer, in order to economize space, designates that form of disease which supervenes within nine days after the injury as acute, and that which occurs subsequently as chronic tetanus.

*Aconite.*—Fourteen cases, all chronic; 8 per cent. recovered; none recovered before fourteen days.

*Amputation.*—60 per cent of seventeen cases recovered; four acute; four in less than fourteen days; three chronic cases died, and one after fourteen days.

*Bleeding.*—55 per cent of fifty-eight cases recovered; nine acute, ten before the fourteenth day. Seven chronic cases died and two after fourteen days.

*Calabar Bean.*—39 per cent of recoveries in thirty-nine cases. Only one recovery occurred in the acute form of the disease. In five others recovery took place before the expiration of fourteen days. *Per contra*, ten cases of chronic tetanus proved fatal.

*Chloroform* relieved 70 per cent of thirty-five cases, nine of which were acute and eight recovered before fourteen days. Three chronic cases died, and two after symptoms lasted fourteen days.

*Cold Effusion.*—73 per cent of eleven cases recovered; three acute, three before fourteen days. Two chronic cases died.

*Ether.*—6 per cent of fifteen cases recovered; five acute; seven inside of fourteen days. One chronic case died.

*Indian Hemp.*—Used in twenty-five cases with 64 per cent of recoveries, of which three cases were acute and six recovered before the symptoms lasted fourteen days. One chronic case died and one after fourteen days.

*Ice Bags.*—77 per cent of nine cases recovered; one acute; two in less than fourteen days.

*Mercury.*—75 per cent of seventy-five cases got well; light acute; twelve before fourteen days; seventeen chronic cases were lost, and two after fourteen days.

*Opium.*—57 per cent of one-hundred and sixty-five cases recovered; twenty-two acute, twenty-nine before fourteen days. Twenty-six chronic cases were lost and four after the disease had continued fourteen days.

*Purgatives.*—66 per cent of seventy-four cases recovered; thirteen acute, twelve before fourteen were lost and four after the disease had continued fourteen days.

*Quinine.*—73 per cent of fifteen cases recovered; one acute; three before fourteen days. Three chronic cases ended fatally, and one after fourteen days' duration.

*Stimulants.*—8 per cent of thirty-three cases recovered: four acute; six within fourteen days. Six chronic cases died, and three after four days.

*Tobacco* relieved 50 per cent of forty-one cases: six acute; six before fourteen days of disease. Four chronic cases died, and one after fourteen days.

*Turpentine* relieved 70 per cent of sixteen cases: six acute; four before fourteen days. Five chronic cases died and two after fourteen days.

*Division of Nerve* is recorded as giving relief in two of three cases.

*Nerve Stretching*, to which a recent contributor of the *Lancet and Clinic* called attention for the relief of traumatic tetanus, is not included in this table. Dr. Yandall thinks the above facts show that we are not improving in our treatment of tetanus. Chloroform ranks in these tables as the most efficient agent yet known in tetanus; while even mercury and venesection, which belong to a past age, excel the Calabar bean of which so much was expected, not only in general results, but in power of control over the disease in its acute form. An astonishing fact is that in the long list of remedies there are but three—musk—woorara and warm baths, where the per centage of recoveries was less than by Calabar bean; and even here, the first and last of this trio effected an equal number of cures of acute tetanus. The conclusion which a careful review of the tables seems to warrant are summarized as follows:

1. That traumatic tetanus is most fatal during the first decade of life.

2. That it usually supervenes between four and nine days after the injury.

3. That the largest number of recoveries are found in cases in which the disease occurred after the lapse of nine days from the injury.

4. When tetanus continues fourteen days, recovery is the rule and death the exception, *apparently indepenaent of the treatment.*

5. Tetanus arising during the puerpral state is the most fatal form of the disease.

6. Chloroform has up to this time yielded the largest percentage of cures in acute tetanus.

7. The true test of a remedy for tetanus is its influence on the history of the disease: (*a*) Does it cure cases in which the disease occurred prior to the ninth day after the injury? (*b*) Does it fail in cases whose duration exceeds fourteen days?

8. Tried by these tests no agent has yet established its claims as a true remedy for tetanus.

